# Localization and Mobility of Synaptic Vesicles in Myosin VI Mutants of *Drosophila*

**DOI:** 10.1371/journal.pone.0102988

**Published:** 2014-07-25

**Authors:** Marta Kisiel, Kristopher McKenzie, Bryan Stewart

**Affiliations:** 1 Department of Biology, University of Toronto Mississauga, Mississauga, Canada; 2 Department of Cell and Systems Biology, University of Toronto, Toronto, Canada; Columbia University, United States of America

## Abstract

**Background:**

At the *Drosophila* neuromuscular junction (NMJ), synaptic vesicles are mobile; however, the mechanisms that regulate vesicle traffic at the nerve terminal are not fully understood. Myosin VI has been shown to be important for proper synaptic physiology and morphology at the NMJ, likely by functioning as a vesicle tether. Here we investigate vesicle dynamics in Myosin VI mutants of *Drosophila*.

**Results:**

In *Drosophila*, Myosin VI is encoded by the gene, *jaguar (jar)*. To visualize active vesicle cycling we used FM dye loading and compared loss of function alleles of *jar* with controls. These studies revealed a differential distribution of vesicles at the *jar* mutant nerve terminal, with the newly endocytosed vesicles observed throughout the mutant boutons in contrast to the peripheral localization visualized at control NMJs. This finding is consistent with a role for Myosin VI in restraining vesicle mobility at the synapse to ensure proper localization. To further investigate regulation of vesicle dynamics by Myosin VI, FRAP analysis was used to analyze movement of GFP-labeled synaptic vesicles within individual boutons. FRAP revealed that synaptic vesicles are moving more freely in the *jar* mutant boutons, indicated by changes in initial bleach depth and rapid recovery of fluorescence following photobleaching.

**Conclusion:**

This data provides insights into the role for Myosin VI in mediating synaptic vesicle dynamics at the nerve terminal. We observed mislocalization of actively cycling vesicles and an apparent increase in vesicle mobility when Myosin VI levels are reduced. These observations support the notion that a major function of Myosin VI in the nerve terminal is tethering synaptic vesicles to proper sub-cellular location within the bouton.

## Background

Although new synaptic vesicles are known to be transported to the nerve terminal from the cell body along microtubule tracks, less is known about the regulation of vesicle traffic within the nerve terminal itself. It was previously thought that upon delivery to the nerve terminal, synaptic vesicles remained relatively static until they were mobilized for neurotransmitter release [1], [2]. This brief period of free mobility was attributed to disassembly of the actin cytoskeleton, which was thought to otherwise cage the vesicles when the synapse was at rest [3]. However, recent work has shown that, even at rest, vesicles are mobile within the synaptic bouton. Fluorescently tagged synaptic vesicles in unstimulated *Drosophila* NMJs exhibited rapid recovery times following photobleaching [4]. Likewise, synaptic vesicles at goldfish ribbon synapses were shown to be highly mobile and this mobility is not related to changes in calcium concentration or the actin cytoskeleton [5].

Myosins are a superfamily of actin-based motor proteins that use energy derived from ATP hydrolysis to move along actin filaments [6]. Myosin VI, first identified in *Drosophila melanogaster*, shares the well-conserved basic structural conformation of other Myosin proteins [7]. However, Myosin VI exhibits a unique reverse directionality; it is the only myosin known to move towards the minus or pointed ends of actin filaments [8]. A role for Myosin VI in regulating the synaptic vesicle localization has been implicated in mammalian cells, where Myosin VI has been shown to associate with endocytic vesicles following clathrin uncoating and to subsequently transport these uncoated vesicles through the actin-rich periphery to the early endosome [9]. Additionally, we have shown previously that at the *Drosophila* NMJ Myosin VI plays an important role in maintaining proper peripheral vesicle localization within the nerve terminal [10]. Myosin VI mutants of *Drosophila* also exhibit impaired neurotransmission, consistent with a function of Myosin VI in tethering vesicles to the bouton periphery [10]. The disruption in vesicle localization, taken together with the defects in synaptic transmission present in mutant larvae, suggests that Myosin VI may participate in mediating synaptic vesicle mobility at the synaptic bouton.

The present study was undertaken to further investigate the role of Myosin VI in synaptic vesicle localization and mobility. Two *in vivo* imaging methods were used to investigate intra-bouton synaptic vesicle localization and mobility at the *Drosophila* third instar larval NMJ: FM dye labeling and fluorescence recovery after photobleaching (FRAP). FM dye labeling revealed vesicle mislocalization of actively cycling vesicles following stimulation in the nerve terminals of Myosin VI mutants, consistent with previous Synaptotagmin labeling of fixed specimens. We also show, by way of FRAP analysis, that a reduction in Myosin VI expression corresponds to an increase in synaptic vesicle mobility. These data lend strong support to the idea that Myosin VI acts as an anchor to restrict vesicles in *Drosophila* boutons and ensure proper vesicle localization and trafficking.

## Methods

### Fly Genetics

All fly strains and crosses were maintained on Bloomington standard medium (http://flystocks.bio.indiana.edu/bloom-food.htm) supplemented with yeast paste at room temperature. The Myosin VI loss of function alleles used in this study were *jar^322^* and *Df(3R)crb87-5* (maintained as stocks over *Tm3, Sb Ser GFP*). Oregon R (*OreR*) flies served as wild-type controls. During handling, flies were temporarily anesthetized using CO_2_. To visualize synaptotagmin dynamics *in vivo*, control *elav^C155^Gal4; UAS-synaptotagmin-GFP* flies were used and were crossed to *jar* mutant genotypes. *elav^C155^Gal4; UAS-synaptotagmin-GFP* stocks (from herein abbreviated to C155SytGFP) contain the nervous system Gal4 driver, *elav^C155^Gal4*, and a *UAS-synaptotagmin-GFP* transgene in the same fly [11].

### FM Dye Labeling

To visualize FM dye uptake during electrophysiological stimulation, experiments were performed on a electrophysiology station equipped with a fluorescent microscope. Wandering third instar larvae were dissected in HL3 solution on Sylgard-coated glass slides. Intracellular recordings were taken from muscles 6 and 7 using a reference electrode filled with a 3 M solution of KCl. An AxoClamp 2B amplifier (Axon Instruments) was used to make recordings. All muscles used for analysis had a resting potential of at least −50 mV. To ensure release of the reserve pool of vesicles, high frequency stimulation was performed at 10 Hz for 10 minutes. During the first stimulation protocol, larval fillets were bathed in HL3 solution with 1.5 mM Ca^2+^ containing the FM1-43 dye at a concentration of 4 µM. During the second stimulation protocol, larval fillets were bathed in 2 mM Ca^2+^ containing with the FM1-43 dye at a concentration of 10 µM. FM dye labeling at NMJ 6/7 of third instar larvae was imaged using a using a 60× water immersion lens on a Nikon E600FN fluorescent microscope fitted with a Hamamatsu Orca CCD camera. The microscope filter was set for GFP emission and excitation.

### Fluorescence Recovery after Photobleaching

Experiments were performed on third instar larval fillets glued to Slygard-coated glass slides in HL3 saline. All images were acquired within 2 hours from the time of dissection. Larvae were glued to plates using a topical tissue adhesive (GLUture, Abbott Laboratories). Recordings of vesicle dynamics were taken from type Ib boutons on NMJ 6/7, with a maximum of 6 boutons from the same larvae used. For the first FRAP protocol, a total of 60 images were taken over a period of 2 minutes, with a 1 second delay between image acquisition. Images were captured as 512×512-pixel frames with the 488-nm line of the argon laser on an LSM510 (Carl Zeiss) confocal laser microscope. These images consisted of four baseline images taken prior to bleaching at 5% of full laser power, followed by the fifth scan consisting of 9 rapid iterations of bleaching at 97% of full laser power in the selected region of interest (ROI). The remaining 56 scans were then completed at 5% of full laser power. In a second series of experiments the protocol was adjusted to capture additional data points for fluorescence recovery immediately following bleaching. In this protocol, a total of 45 images were taken over a period of 45 seconds with no delay between image acquisition. Images were captured as 256×256-pixel frames with the 488-nm line of the argon laser on an LSM510 (Carl Zeiss) confocal laser microscope. Again, these images consisted of four baseline images taken prior to bleaching at 5% of full laser power, followed by the fifth scan consisting of 12 rapid iterations of bleaching at 97% of full laser power in the selected ROI. The remaining 41 scans were then completed at 5% of full laser power. Constant imaging parameters were maintained between samples and the size of the ROI was kept constant at 24×30 pixels in both FRAP protocols.

Fluorescence intensity was measured in Image J for the bleached region, the background and the total bouton area using the Time Series Analyzer plug-in. To account for photobleaching during the experiment and the movement of bleached molecules, recovery curves were generated following double normalization of the raw fluorescence intensity [12]. Average fluorescence intensity was measured at each time point during imaging for the bleached region (ROI), the whole bouton (WB) and the region outside the bouton (BG). This calculation is summarized in the following equation:
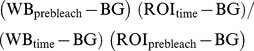



Differences between recovery curves were assessed by fitting the data with a double exponential curve. A double exponential curve was used to account for the two phases in fluorescence recovery observed with respect to vesicle mobility, the initial more rapid recovery immediately post bleaching and the later plateau [13].

### Statistical Analysis

All statistical analyses were performed in GraphPad Prism Software 5. A significance level of p<0.05 was used for all experiments.

## Results

### Vesicle distribution is altered in Myosin VI mutants following high frequency stimulation

We previously reported on mislocalization of synaptic vesicles in Myosin VI mutants of *Drosophila*, via immunofluorescent detection of Synaptotagmin (Kisiel et al., 2011). Here we seek to investigate vesicle localization and dynamics in living specimens.

**Figure 1:**
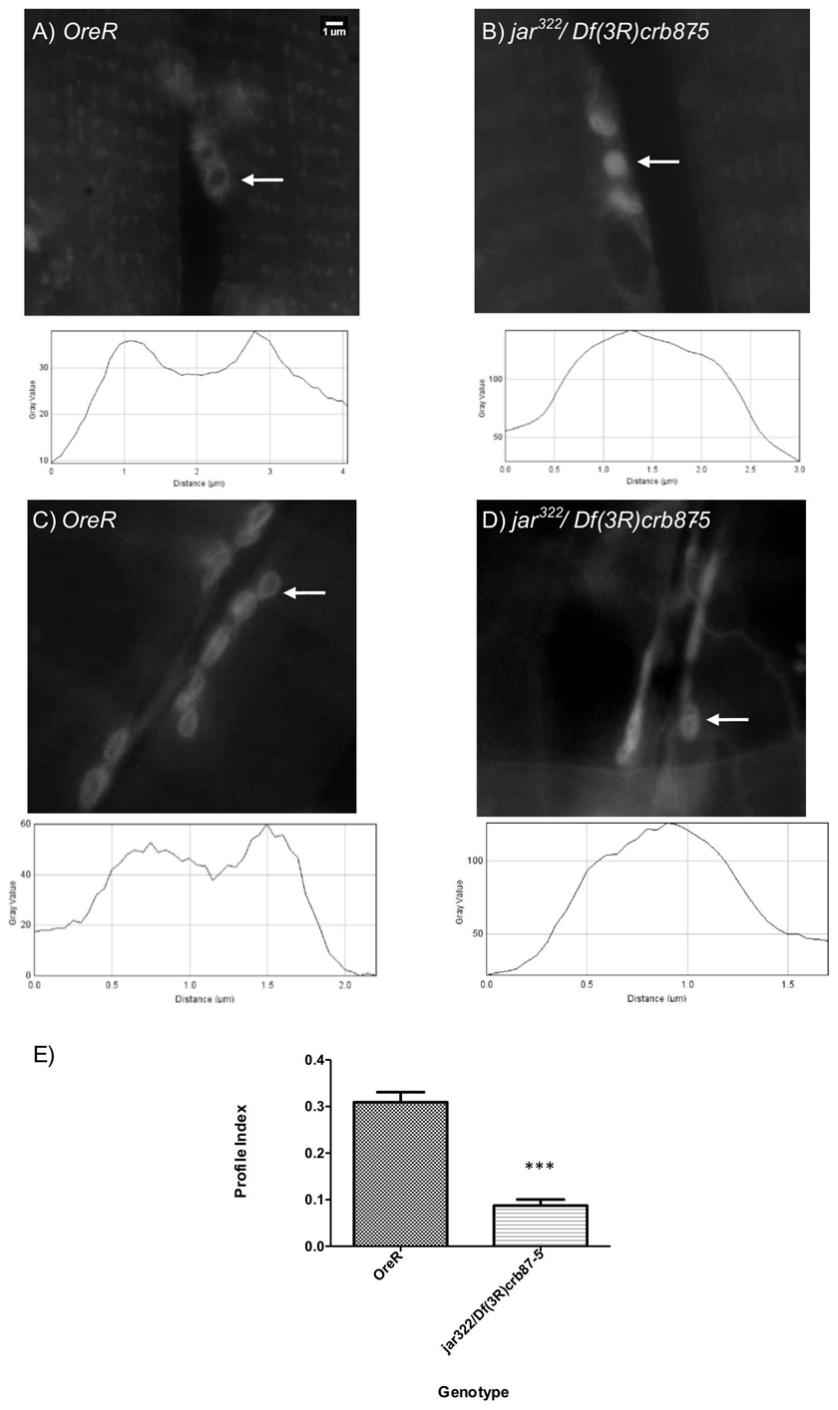
FM1-43 dye labeling reveals differential synaptic vesicle distribution in Myosin VI mutant boutons. Two representative images showing the distribution of FM labeled synaptic vesicles with their corresponding plot profiles for control *OreR* (A) and *jar^322^/Df(3R)crb87-5* mutant larvae (B). Arrows highlight an Ib bouton in the control with peripheral FM dye localization and an Ib bouton in the *jar* mutant with diffuse FM dye localization. Boutons were labeled with a concentration of 4 µM FM 1-43 dye in 1.5 mM Ca^2+^ saline. There was a significant reduction in relative difference in fluorescence intensity across *jar^322^/Df(3R)crb87-5* mutant boutons (n = 7) compared to the control boutons (n = 6; Unpaired T-test, *** = p<0.001). ). Boutons used for this study were from muscle 6/7 NMJs in segments A3, A4 and A5.

FM dye labeling was used to examine synaptic vesicle distribution *in vivo* following 10 Hz nerve stimulation for 10 minutes. Boutons were labeled with two concentrations of FM 1–43 dye, 4 µM and 10 µM, in 1.5 mM and 2 mM Ca^2+^ HL3 saline respectively. Differences in FM dye uptake were quantified by measuring the relative difference in fluorescence intensity across a bouton's plot profile and calculating a profile index [(max intensity-min intensity)/max intensity]. For the first labeling protocol using 4 µM dye and 1.5 mM calcium saline, the characteristic plot profile for *OreR* boutons has two peaks indicating a peripheral distribution of FM labeled vesicles (Figure 1). In contrast, a single maxima is observed extending across the entirety of the bouton for *jar^322^/Df(3R)crb87-5* mutant larvae indicating a diffuse distribution of FM labeled vesicles (Figure 1). We found the profile index for fluorescence intensity was significantly smaller for jar322*/Df(3R)crb87-5* mutant boutons compared to *OreR* control boutons (Unpaired T-test, p<0.001). There was no significant difference in mean fluorescence across the entire bouton between *OreR* controls and *jar^322^/Df(3R)crb87-5* mutant larvae (Unpaired T-test, p>0.05).

**Figure 2:**
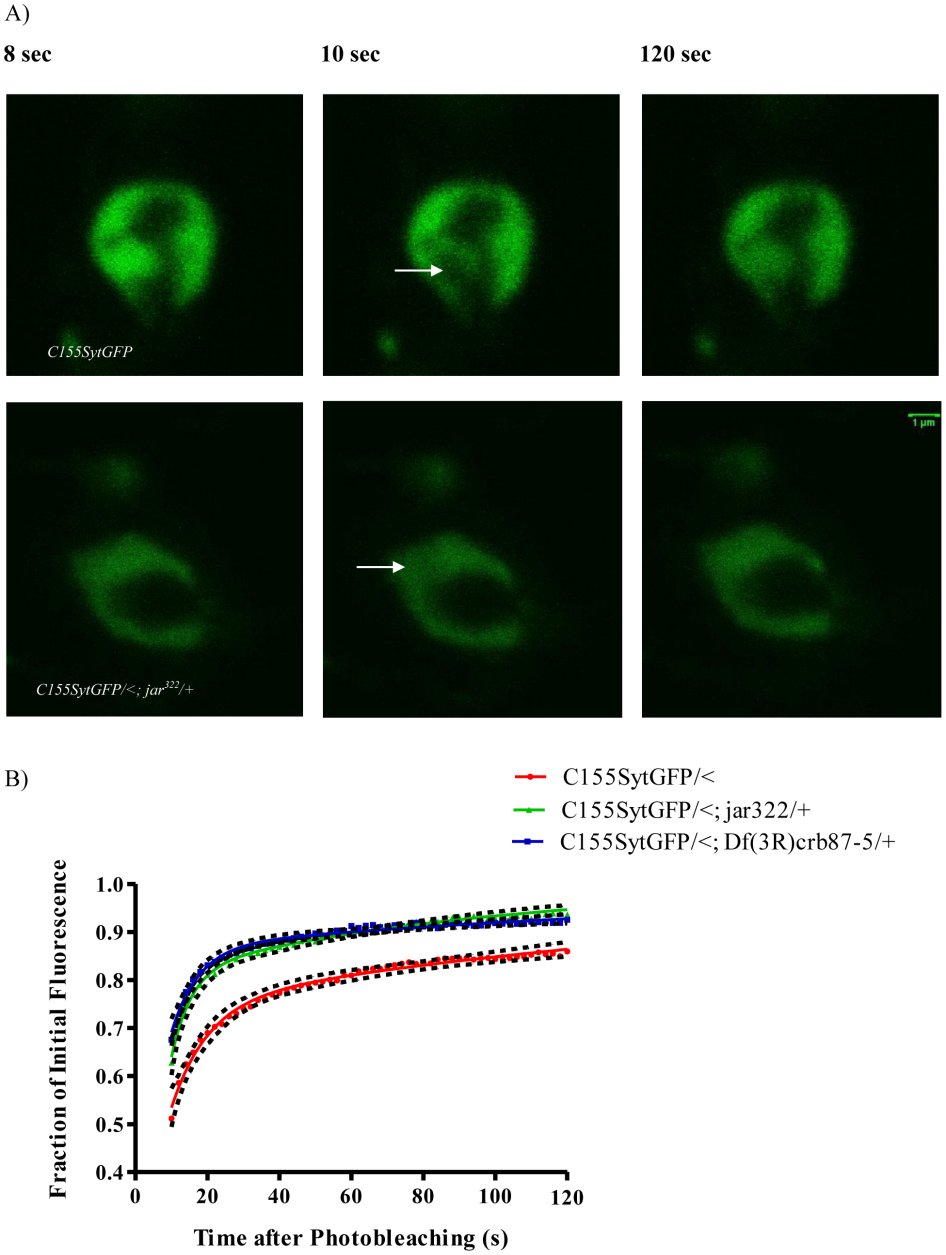
Further FM1-43 dye labeling highlights diffuse synaptic vesicle distribution in Myosin VI mutant boutons. Two representative images showing the distribution of FM labeled synaptic vesicles with their corresponding plot profiles for control *OreR* (A) and *jar^322^/Df(3R)crb87-5* mutant larvae (B). Arrows highlight an Ib bouton in the control with peripheral FM dye localization and an Ib bouton in the *jar* mutant with diffuse FM dye localization. Boutons were labeled with a concentration of 10 µM FM 1-43 dye in 2 mM Ca^2+^ saline. A significant reduction in relative difference in fluorescence intensity was observed across *jar^322^/Df(3R)crb87-5* mutant boutons (n = 34) compared to the control boutons (C; n = 28; Unpaired T-test, *** = p<0.001). Boutons used for this study were from muscle 6/7 NMJs in segments A3, A4 and A5.

The second set of labeling experiments was conducted with the higher concentration FM dye and Ca^2+^ solution with the same 10 Hz, 10 minutes stimulation protocol. Similarly, this protocol revealed that the *jar^322^/Df(3R)crb87-5* mutant larvae exhibited a greater proportion of boutons with diffuse FM dye labeling throughout the bouton area in contrast to *OreR* control boutons. Calculating the plot profile index of fluorescence intensity under these conditions also revealed a significantly lower index for *jar^322^/Df(3R)crb87-5* mutant boutons (n = 34) compared to *OreR* control boutons (n = 28, Unpaired T-test, p<0.05). Again, this is consistent with the majority of *jar^322^/Df(3R)crb87-5* mutant boutons exhibiting the diffuse synaptic vesicle distribution phenotype observed as a single maxima when a plot profile is made from the bouton area (Figure 2).

Together these FM-dye experiments reveal that synaptic vesicles occupy a broad domain within the Myosin VI mutant boutons, whereas in controls larvae the vesicles are found within a narrower domain close to the bouton periphery.

### Myosin VI mutants have enhanced synaptic vesicle mobility

To examine vesicle dynamics at *jar* mutant synapses, synaptic vesicles were visualized *in vivo* using a Synaptotagmin-GFP fusion protein and vesicle mobility was assessed using FRAP. Using image acquisition parameters we previously established for FRAP analysis (Nunes et al., 2006), we compared FRAP recovery curves and found a significant increase in synaptic vesicle mobility in the *jar* mutant boutons.

**Figure 3:**
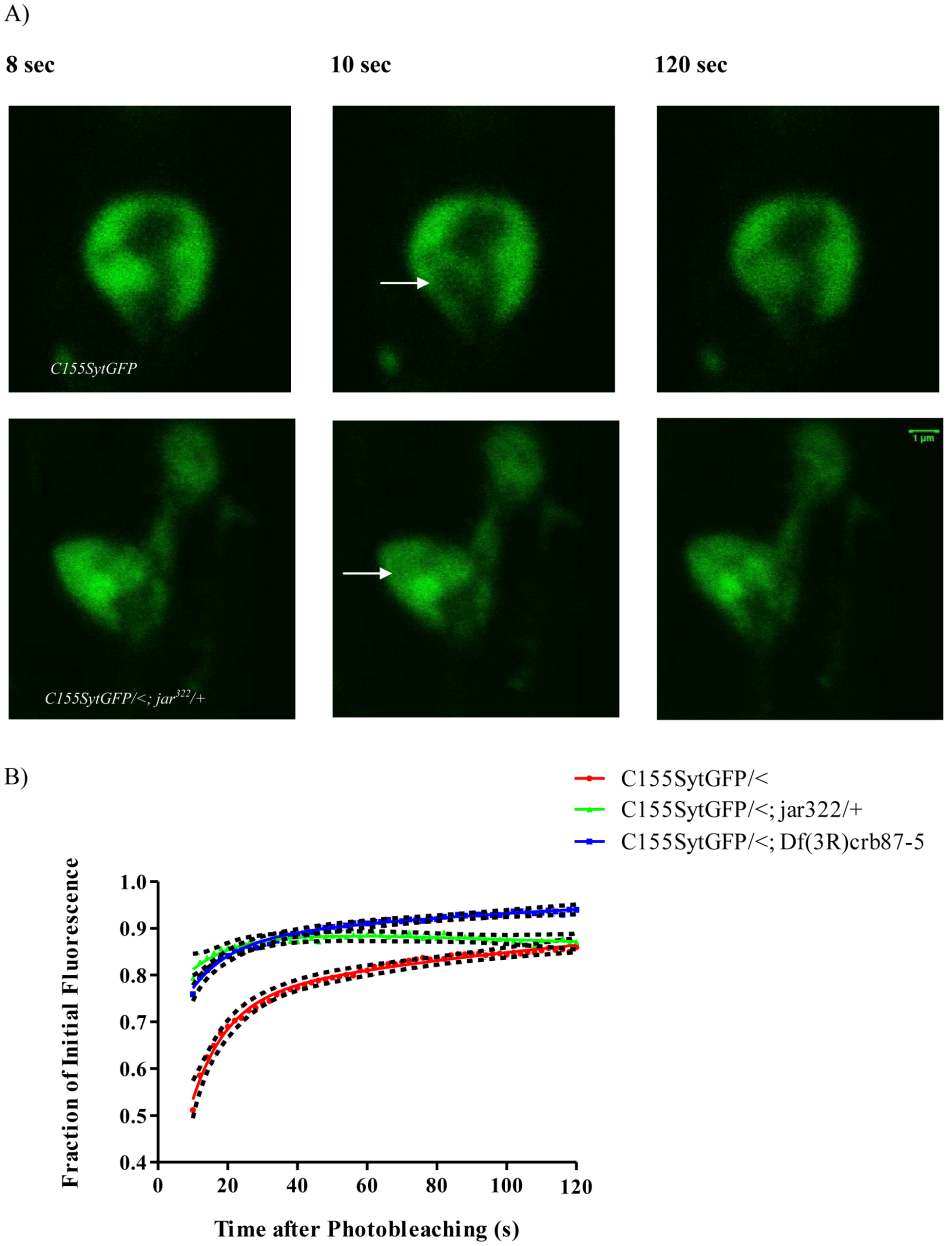
Synaptic vesicle mobility was enhanced in normal *jaguar* mutant boutons. Representative images acquired during FRAP experiments for control and *jar* mutant boutons with proper peripheral vesicle localization revealed more rapid recovery of labeled vesicles into the bleach area of the *jar* mutant bouton (A). Images shown correspond to the time before bleaching (8 sec), immediately after bleaching (10 sec) and at the end of image acquisition (120 sec). The bleached region is indicated by a white arrow. Curve fitting of FRAP recoveries was performed using double exponential curves and statistical differences were tested using nonlinear regression (B). The heterozygous *jar* mutants, *elav^C155^Gal4; UAS-synaptotagmin-GFP/<; jar^322^/+* (n = 18 boutons) and *elav^C155^Gal4; UAS-synaptotagmin-GFP/<; Df(3R)crb87-5/+* (n = 18 boutons), exhibited significantly enhanced vesicle mobility compared to the control *elav^C155^Gal4; UAS-synaptotagmin-GFP/<* (n = 25 boutons; Comparison of Fits, p<0.0001). Boutons used for this study were from muscle 6/7 NMJs in segments A3 and A4. Error is represented as the 95% confidence interval of the curve.

Since we documented changes in vesicle localization in the mutant alleles, as a first step we compared boutons with relatively normal appearance in heterozygous *jar* mutants to controls. Interestingly, we found that heterozygous *jar* mutants, *elav^C155^Gal4; UAS-Synaptotagmin-GFP/<; jar^322^/+* (n = 18 boutons) and *elav^C155^Gal4; UAS-Synaptotagmin-GFP/<; Df(3R)crb87-5/+* (n = 18 boutons), exhibited a greater extent of fluorescence recovery compared to the control, *elav^C155^Gal4; UAS-Synaptotagmin-GFP/<* (n = 25 boutons; Comparison of fits, p<0.0001, Figure 3). Thus, even though vesicle localization appeared ‘normal’ in these heterozygous *jar* alleles, we found evidence for enhanced mobility.

**Figure 4:**
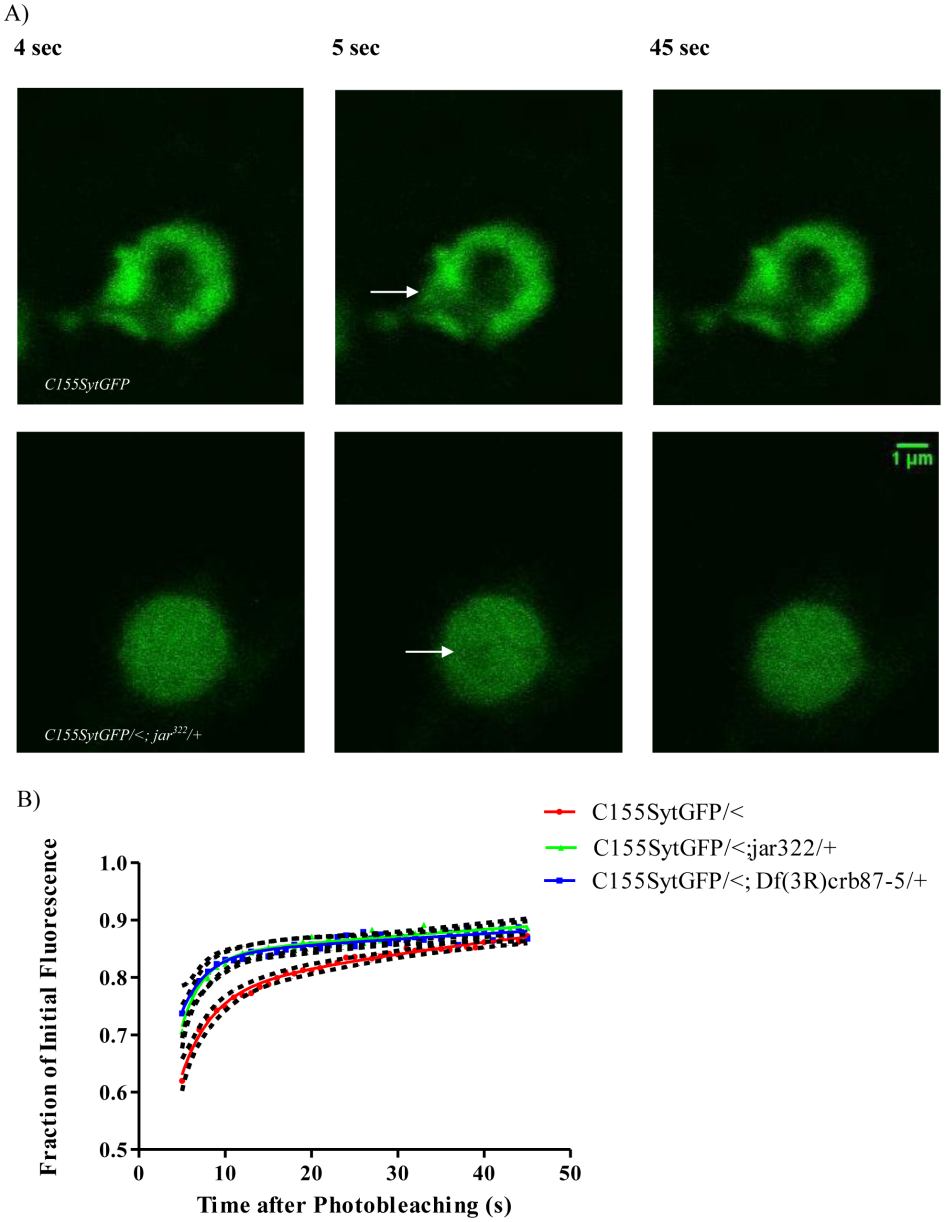
Diffuse *jaguar* mutant boutons exhibited a significant increase in vesicle mobility. Representative images acquired during FRAP experiments for control and *jar* mutant boutons with diffuse vesicle localization revealed more rapid recovery of labeled vesicles into the bleach area of the *jar* mutant bouton (A). Images shown correspond to the time before bleaching (8 sec), immediately after bleaching (10 sec) and at the end of image acquisition (120 sec). The bleached region is indicated by a white arrow. Curve fitting of FRAP recoveries was performed using double exponential curves and statistical differences were tested using nonlinear regression (B). Vesicle mobility was significantly greater in the diffuse boutons of the heterozygous *jar* mutants, *elav^C155^Gal4; UAS-synaptotagmin-GFP/<; jar^322^/+* (n = 20 boutons) and *elav^C155^Gal4; UAS-synaptotagmin-GFP/<; Df(3R)crb87-5/+* (n = 15 boutons), compared to the control *elav^C155^Gal4; UAS-synaptotagmin-GFP/<* (n = 25 boutons; Comparison of Fits, p<0.0001). Boutons used for this study were from muscle 6/7 NMJs in segments A3 and A4. Error is represented as the 95% confidence interval of the curve.

Next we turned to analyze vesicle mobility in boutons with diffuse vesicle distribution. Remarkably, we found an even greater enhancement in vesicle mobility in the diffuse boutons of the heterozygous *jar* mutants, *elav^C155^Gal4; UAS-Synaptotagmin-GFP/<; jar^322^/+* (n = 20 boutons) and *elav^C155^Gal4; UAS-Synaptotagmin-GFP/<; Df(3R)crb87-5/+* (n = 15 boutons), compared to the control *elav^C155^Gal4; UAS-Synaptotagmin-GFP/<* (n = 25 boutons; Comparison of fits, p<0.0001, Figure 4).

**Figure 5:**
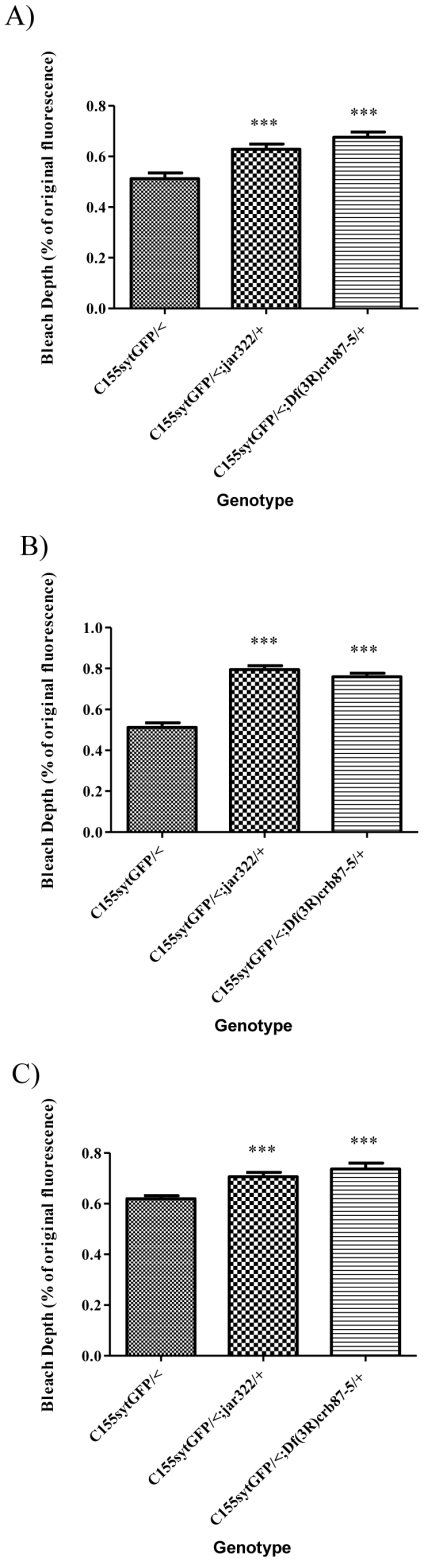
FRAP curves for diffuse *jaguar* mutant boutons have greater initial fluorescence recovery than control boutons. Representative images acquired during FRAP experiments for control and *jar* mutant boutons with diffuse vesicle localization revealed more rapid recovery fluorescence into the bleach area of the *jar* mutant bouton (A). Images shown correspond to the time before bleaching (4 sec), immediately after bleaching (5 sec) and at the end of image acquisition (45 sec). The bleached region is indicated by a white arrow. Curve fitting of FRAP recoveries was performed using double exponential curves and statistical differences were tested using nonlinear regression (B) Rapid fluorescence recovery observed for the FRAP curves of the diffuse boutons of the heterozygous *jar* mutants, *elav^C155^Gal4; UAS-synaptotagmin-GFP/<; jar^322^/+* (n = 15 boutons) and *elav^C155^Gal4; UAS-synaptotagmin-GFP/<; Df(3R)crb87-5/+* (n = 16 boutons), indicates enhanced vesicle mobility compared to the control *elav^C155^Gal4; UAS-synaptotagmin-GFP/<* (n = 35 boutons; Comparison of Fits, p<0.0001). Boutons used for this study were from muscle 6/7 NMJs in segments A3 and A4. Error is represented as the 95% confidence interval of the curve.

In such image sequences, we noticed that there was less bleaching in the mutant genotypes compared to controls. This observation is also consistent with more freely moving vesicles in the mutants. We therefore adjusted our acquisition protocol to account for this difference by employing a greater number of bleach iterations and more rapid image acquisition, to capture additional information about fluorescence recovery in the first part of the recovery curve. With this protocol we confirmed that the diffuse boutons of the *jar* heterozygotes, *elav^C155^Gal4; UAS-Synaptotagmin-GFP/<; jar^322^/+* (n = 15 boutons) and *elav^C155^Gal4; UAS-Synaptotagmin-GFP/<; Df(3R)crb87-5/+* (n = 16 boutons, Comparison of fits, p<0.0001, Figure 5) exhibit greater fluorescence recovery than with the control *elav^C155^Gal4; UAS-Synaptotagmin-GFP/<* boutons (n = 35 boutons).

**Figure 6:**
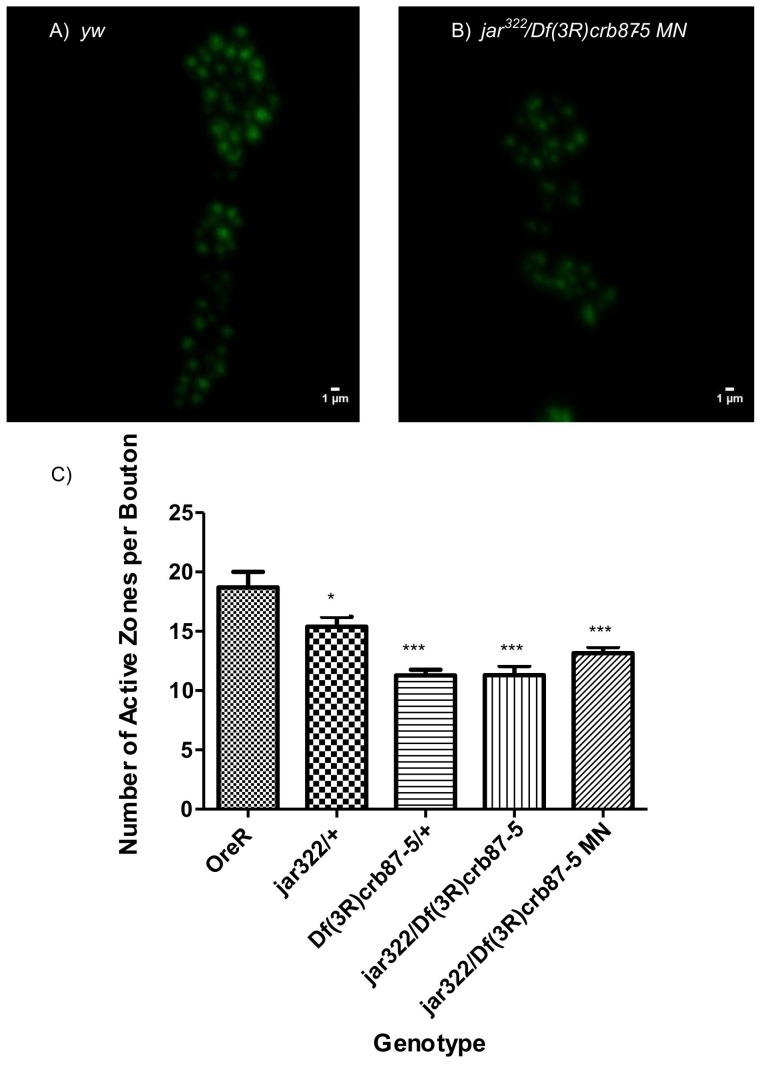
Control boutons exhibit a significantly greater bleach depth than *jar* mutant boutons. Bleach depth is significantly greater for *elav^C155^Gal4; UAS-synaptotagmin-GFP/<* boutons than both the boutons with proper peripheral vesicle localization (A) and the diffuse boutons of heterozygous *jar* mutants immediately following 9 bleach iterations (B, Dunnett's Multiple Comparison Test, *** = p<0.001). Similarly, following 12 bleach iterations, bleach depth was significantly greater for control *elav^C155^Gal4; UAS-synaptotagmin-GFP/<* boutons compared to diffuse boutons of *jar* loss of function heterozygotes (C, Dunnett's Multiple Comparison Test, *** = p<0.001). Boutons used for this study were from muscle 6/7 NMJs in segments A3 and A4. Bars represent mean ± SEM.

Bleach depth was found to be significantly greater for control boutons immediately following 9 bleach iterations compared to the boutons of *jar* heterozygotes with normal and diffuse vesicle distribution (Figure 6, Dunnett's Multiple Comparison Test, p<0.001). Similarly, for the rapid image acquisition FRAP protocol with 12 bleach iterations, bleach depth was significantly greater for control *elav^C155^Gal4; UAS-synaptotagmin-GFP/<* boutons compared to diffuse boutons of *jar* loss of function heterozygotes (Figure 6, Dunnett's Multiple Comparison Test, p<0.001).

## Discussion

### FM dye distribution is altered in Myosin VI mutant boutons following high frequency stimulation

Ultrastructural studies have reliably shown that synaptic vesicles have a peripheral distribution within *Drosophila* type Ib boutons and that the center of the bouton is relatively free of vesicles [14]–[16]. FM1-43 dye loading at low frequency stimulation has been consistent with EM data in showing peripheral vesicle localization following endocytosis [17]. Additionally, FM1-43 dye labeling within the *Drosophila* larval synaptic terminal has confirmed that vesicles in different functional pools are spatially intermixed at the bouton periphery [18]. Centrally localized vesicles can be observed in Ib boutons following FM1-43 dye staining at 10 Hz followed by a 10 minute resting period, which is attributed to the formation of extra vesicles in response to intense stimulation [17]. However, when vesicle localization was visualized immediately following stimulation with no rest period, vesicles were found to occupy a smaller, peripherally area of the bouton [17]. Thus, the redistribution of extra vesicles to the bouton centre occurs during the rest period. Fluorescence intensity following FM1-43 dye loading also provides further information about the vesicle population as it is proportional to the number of vesicles within the nerve terminal [19], [20].

In *jar* alleles of *Drosophila*, FM1-43 dye uptake was induced through a high frequency nerve stimulation protocol using two different dye concentrations. Imaging of vesicle distribution immediately following electrical stimulation and washing of dye revealed that vesicles were localized throughout the bouton area in *jar* loss of function mutant boutons while they remained restricted to the bouton periphery in wild-type controls. Given that FM dye labeling was visualized immediately following high frequency loading and that total bouton fluorescence did not differ between mutants and controls, the central localization of vesicles in *jar* mutants cannot be attributed to an increase in vesicle abundance. Rather, it is suggestive of an active role of Myosin VI in peripheral vesicle clustering at the nerve terminal.

This result is consistent with our previously published Synaptotagmin staining of fixed tissue, which indicated that Myosin VI is important for proper vesicle localization within the bouton. The present FM labeling reveals that actively cycling vesicles also have altered distribution in *jar* mutants and suggest that Myosin VI plays an active role to maintain peripheral vesicle distribution during neural activity.

### Myosin VI loss of function mutants exhibit enhanced vesicle mobility at the synaptic terminal

Synaptic vesicles are mobile at the *Drosophila* NMJ [13], [21]; however, the mechanisms regulating vesicle dynamics at the nerve terminal have yet to be fully resolved. Understanding the mechanisms by which vesicle movement is mediated within nerve terminals can provide insights into the mobilization of vesicles for participation in exocytosis. Vesicles may move within the synaptic bouton by diffusion or via active transport; although the relative contribution of these processes in vesicle mobility is unknown. In frog motor nerve terminals, vesicle motion appears to be mediated by simple diffusion and is not affected by disruption of the actin cytoskeleton [22]. However, other studies indicate that a myosin motor moving along actin tracks may be important for regulating vesicle mobility [13], [23].

If Myosin VI functions in synaptic vesicle localization to the bouton periphery by acting as a tether, it should normally restrain vesicle movement at the synapse. To test that idea, we undertook FRAP analysis of vesicle movement in the living nerve terminal of the *jar* alleles. FRAP analysis revealed a significant enhancement of vesicle mobility in *jar* mutant boutons that showed both normal and diffuse vesicle distribution phenotypes. However, the enhancement in vesicle mobility compared to the controls was even greater for diffuse boutons than the *jar* boutons with proper torus-shaped localization of vesicles. In addition, bleach depth was significantly less for mutant boutons compared to controls, which is indicative of fluorescence recovery occurring so rapidly it is taking place during bleaching itself [24]–[26]. This finding is interesting given that disruption of the actin cytoskeleton itself reduces vesicle mobility at the *Drosophila* nerve terminal [4]. With Myosin VI being an actin-based motor, it may be expected that loss of Myosin VI would likewise result in reduction in vesicle mobility. We did not make such an observation. However, it is possible that Myosin VI performs its anchoring function through an interaction with the microtubule cytoskeleton via adaptor proteins. Assays to detect protein interactions show that Myosin VI binds to the microtubule associated protein Cornetto [27]. In addition, Myosin VI has been shown to co-immunoprecipitate with and co-localize with the microtubule-binding protein CLIP-190 in the *Drosophila* embryonic nervous system [28]. Thus, the FRAP results are consistent with a function for Myosin VI as a vesicle tether because *jar* mutants have a lower level of Myosin VI expression and therefore, a reduced ability to anchor vesicles resulting in their enhanced mobility at the synapse. The FRAP data is important because it provides the mechanism of Myosin VI function at the synapse. Myosin VI may maintain proper synaptic physiology by tethering vesicles to ensure their proper localization for efficient release.

Correct vesicle organization is important for subsequent steps in neurotransmitter release following neural stimulation. What is the relationship of these vesicle mobility results to the known physiological phenotypes of *jar* synapses? We previously reported that there was only a detectable change in low-frequency synaptic strength, observed as a ∼⅓ reduction in EJP amplitude and a small reduction in mEJP frequency, in the most severe combination of *jar* alleles. Whereas in this report we show that there are measurable changes in FRAP recovery, even in the milder *jar* alleles. Together these data suggest that the process of synaptic transmission is relatively insulated from the intermediate increase in vesicle mobility we observe here with the mild *jar* alleles, but that the greatest increase in mobility correlates with a modest decrease in synaptic strength. This result implies that there may be a small reduction in the immediately releasable pool of vesicles in the *jar* mutant. These data also show that FRAP measurements are more sensitive for observing phenotypic changes in vesicle physiology, than EJP measurements alone.

In addition, we previously demonstrated that high-frequency stimulation paradigms revealed enhanced frequency-dependent increases in synaptic strength. At 10 Hz facilitation, severe *jar* alleles showed greater increases in synaptic transmission in 1 mM Ca^++^ and a greater extent of synaptic depression in 10 mM Ca^++^ than either control or less severe *jar* combinations, which behaved similarly. This observation indicates that although vesicles are moving rapidly throughout the bouton they can transition from the reserve pool to the readily releasable for participation in synaptic transmission and indeed appear to be able to sustain high rates of transmitter release. Although the mechanisms that regulate the translocation of vesicles between different functional pools during high frequency stimulation have not been fully resolved, Myosin motors have been implicated in this function in other preparations [29], [30]. Altogether, these data suggest that removal of Myosin VI leads to greater vesicle mobility and that those vesicles are available for transmitter release. The most severe *jar* alleles lead to concomitant increased vesicle mobility, reduced synaptic output at low frequencies of stimulation and enhanced plasticity at higher frequencies of stimulation.

## Conclusions

In summary, the present work shows that Myosin VI plays a critical role in restraining synaptic vesicles to the bouton periphery. In the absence of Myosin VI function, peripheral vesicle localization is disrupted and vesicle mobility is enhanced at the synapse. The changes in vesicle mobility that we report here may, in part, account for the physiological changes to synaptic transmission we previously described in Myosin VI loss of function mutants. Future studies aimed at understanding dynamic regulation of Myosin VI function will yield information about the control of synaptic vesicle pools during synaptic transmission.
